# Delivery of DNA‐encoded vaccines and proteins in mice using cutaneous suction‐mediated transfection

**DOI:** 10.1002/2211-5463.70306

**Published:** 2026-07-09

**Authors:** Emran O. Lallow, Isabel Brandtjen, Yaxin Mo, Melissa Gulley, Louis Osorio, Sagar Kudchodkar, Nandita C. Jhumur, Christine C. Roberts, Lisa K. Denzin, David I. Shreiber, Biju Parekkadan, Hao Lin, Joel N. Maslow

**Affiliations:** ^1^ GeneOne Life Science Seoul South Korea; ^2^ Department of Biomedical Engineering Rutgers University‐New Brunswick New Jersey USA; ^3^ Department of Mechanical and Aerospace Engineering Rutgers University‐New Brunswick New Jersey USA; ^4^ Department of Pediatrics, Child Health Institute of New Jersey Rutgers Medical School New Brunswick New Jersey USA; ^5^ Department of Medicine Morristown Medical Center Morristown New Jersey USA; ^6^ Present address: Eurofins Scientific SE Lancaster Pennsylvania USA; ^7^ Present address: Gates Foundation Seattle Washington USA; ^8^ Present address: Zoluxion LLC Seattle Washington USA

**Keywords:** cutaneous suction, DNA vaccination and therapeutics, *In vivo* nucleic acid delivery, preclinical mouse model

## Abstract

A critical limitation of DNA vaccines and other therapeutics is transfection *in vivo* to produce the encoded antigens or therapeutic proteins. Cutaneous suction‐based methods have demonstrated effectiveness in many animal models and have been successfully applied in human clinical trials, but have not been extended to mouse models, where numerous disease models, transgenic strains, and murine‐specific reagents exist. Here, we establish and optimize methods for cutaneous suction‐mediated DNA transfection in mice. By adapting a smaller cup diameter and smaller injection volume, the challenges of skin hyperelasticity and decreased skin thickness can be effectively addressed. Thus, we demonstrate that vaccinating mice with the GLS‐5310 SARS‐CoV‐2 DNA vaccine using this method yields high levels of binding antibody and T‐cell responses. Additionally, suction following injection of a novel pVAX1‐based expression vector yielded systemic levels of a SEAP transgene. We conclude that suction‐mediated delivery of nucleic acid‐based therapies and vaccines can be a valuable tool for the study of preclinical mouse models.

## Graphical abstract

Cutaneous suction‐mediated transfection in mice for delivery of DNA‐encoded vaccines and proteins.

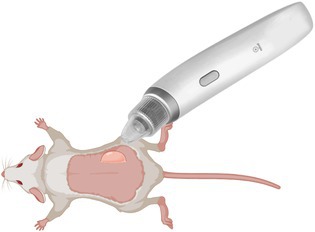

**Abbreviations**
ANOVA: analysis of variance
CMV: cytomegalovirus
DNA: deoxyribonucleic acid
ELISA: enzyme‐linked immunosorbant
ELISPOT: enzyme‐linked immunospot
HaCaT: human adult high calcium low‐temperature keratinocytes
ID: intradermal
IP: intraperitoneal
IVIS: *in vivo* imaging system
ORF3a: opening reading frame 3a
SARS‐CoV‐2: severe acute respiratory syndrome coronavirus 2
SEAP: secreted embryonic alkaline phosphatase
SFU: spot forming units

DNA vaccines and therapeutics have demonstrated great promise due to their stability, efficacy, and cost‐effectiveness [1, 2, 3], with one vaccine, ZyCoV‐D, approved for human use in India [4]. Although several devices have been used to promote *in vivo* transfection of injected DNA, the currently available delivery mechanisms are associated with high cost, tissue damage, and rigorous training requirements [1, 5, 6]. We developed a novel suction‐based delivery device, GeneDerm, that has shown applicability in preclinical animal models, including hamsters, rats, rabbits, and ferrets, and has been successfully applied in human clinical trials of the GLS‐5310 DNA vaccine [7, 8, 9, 10, 11]. The GeneDerm device is used to apply negative pressure suction atop the site of intradermally injected plasmid DNA, which in turn induces efficient *in vivo* DNA transfection. GeneDerm has many advantages as a DNA delivery device, such as being cost‐effective, pain‐free, and requiring minimal user training. To date, GeneDerm has not yet been widely used in mice primarily due to the hyperelasticity of mouse skin and decreased skin thickness as compared to other preclinical animal models; however, others have successfully achieved *in vivo* transfection to liver, heart, spleen, and kidneys of mice when suction is applied directly to the respective solid organ following injection of plasmid DNA [12, 13]. As numerous transgenic strains and murine‐specific reagents exist that are central to the preclinical study of many diseases, extension of this DNA delivery method to mice is highly desirable.

Here, we establish and optimize methods for suction‐mediated DNA transfection in mice. We show that by using a smaller cup diameter and smaller injection volume, we are able to achieve high levels of transfection and transgene expression for DNA vaccines. Additionally, we show that the use of GeneDerm following the injection of a novel pVAX1‐based expression vector yields detectable quantities of expressed proteins in the bloodstream of mice. GeneDerm can be a valuable tool for the study of nucleic acid‐based therapies and vaccines in preclinical mouse models.

## Materials and methods

### Animal preparation and experiments

Six‐ to eight‐week old BALB/c (BALB/cAnNTac) and C57BL/6 (C57BL/6NTac) female mice were purchased from Taconic Biosciences Inc. (Germantown, NY). Mice were housed in micro‐isolator cages and were provided *ad libitum* access to food and water. The housing room was maintained on a regulated 12‐light/12‐h dark cycle, in accordance with the guidelines established by the Rutgers University Institutional Animal Care and Use Committee, under protocol IACUC‐201800077.

**Fig. 1:**
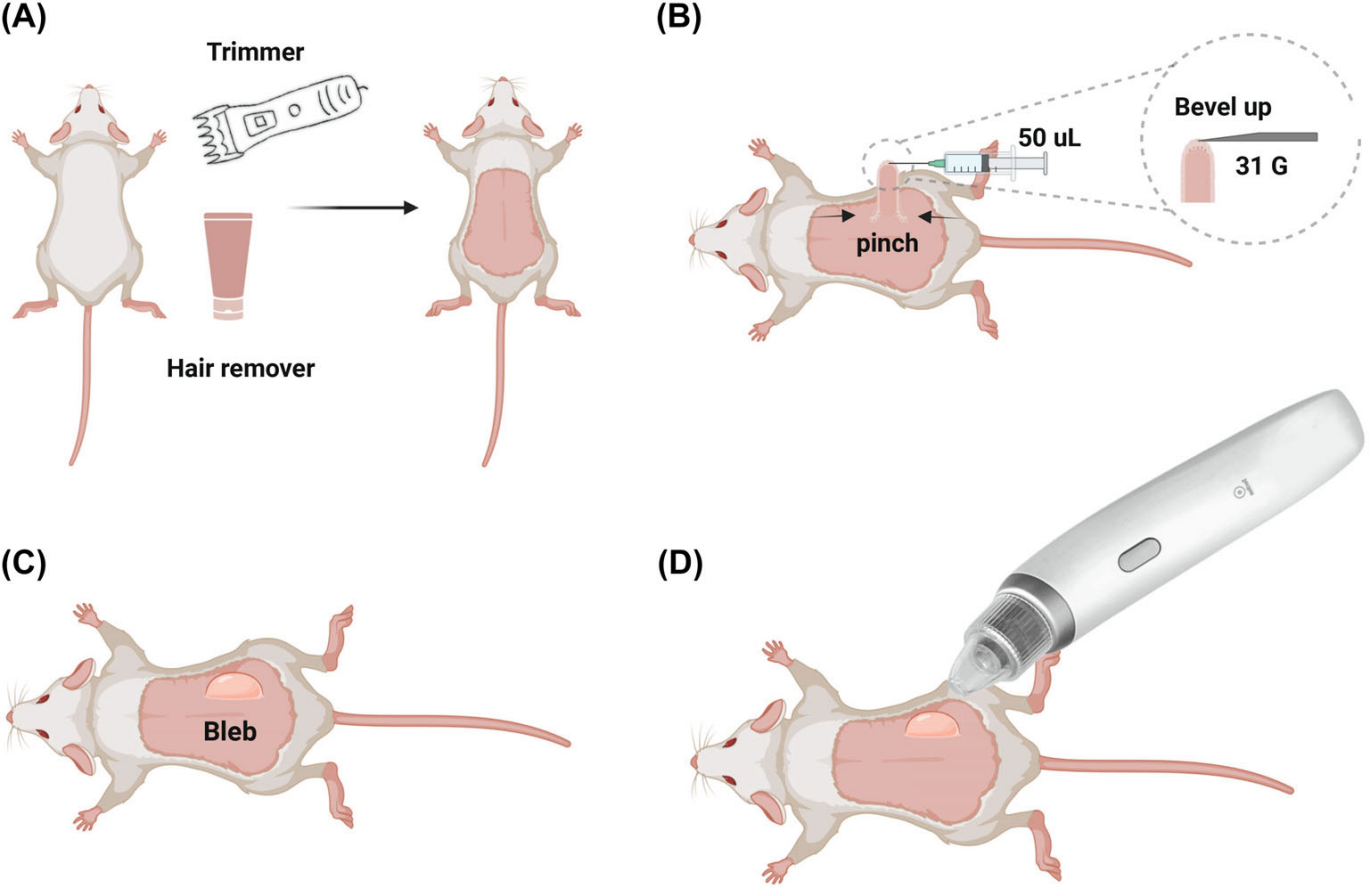
Schematic diagram of the experimental procedure and technique. (A) The hair is removed from the dorsal area. (B) A solution is injected intradermally into pinched skin to form the bleb. (C, D) The application of the negative pressure using the GeneDerm Device atop the bleb. Created in BioRender. Lallow, E. (2026) https://BioRender.com/ay3wk5s.

Preparation of mouse skin for intradermal (ID) injection and subsequent application of suction was performed as previously described for rats and hamsters [10] with the following modifications (Fig. 1). Procedures were performed for mice anesthetized with isoflurane. Following hair removal from the dorsal region by shaving and depilation (Fig. 1A), the skin was pinched with the thumb and forefinger. Intradermal (ID) injection was accomplished with a 31‐gauge SOL‐M needle (SOL‐Millennium, Chicago, IL) bevel upward, parallel to the skin surface, ensuring very shallow injection (Fig. 1B) to yield a postinjection bleb (Fig. 1C) within the mouse skin. Suction is then applied atop the bleb using the GeneDerm device with a cup opening of either 2 mm or 3 mm (Fig. 1D), as noted.

### Luminescence imaging and quantification

Six‐ to eight‐week‐old BALB/c (BALB/cAnNTac) and C57BL/6 (C57BL/6NTac) female mice were injected via the ID route with pGLS‐Luc2 (purchased as pGL4.14, Promega, Madison, WI), a plasmid encoding the firefly luciferase protein. XenoLight D‐Luciferin Potassium Salt (PerkinElmer, Waltham, MA) was injected intraperitoneally (IP) at a dose of 150 mg·kg^−1^ in a 200 μL volume, which served as a substrate to produce bioluminescence when oxidized by luciferase. At 10 min postinjection, animals were placed under anesthesia and imaged using the IVIS Lumina X5 live imaging system (PerkinElmer, Waltham, MA). Luminescence was determined following a 3‐s exposure. The Aura imaging software (Model 4.0.8, Spectral Instruments, Tucson, AZ) was used to quantify the total photon emission at each injection site. Background signal was determined for a nontreated region and then subtracted from the treated regions. A square root transformation was applied to all conditions. Graphical presentation and statistical analysis were performed using graphpad prism (10.2.3; graphpad software, San Diego, CA). At the end of the study, mice were euthanized via CO_2_ inhalation followed by cervical dislocation as a second method to ensure death in accordance with Rutgers IACUC guidelines.

### GLS‐5310 vaccination and immunology

Ten six‐ to eight‐week‐old BALB/c (BALB/cAnNTac) mice were vaccinated with 25 μg of the GLS‐5310 COVID‐19 DNA vaccine, which expresses the SARS‐CoV‐2 spike and ORF3a proteins [7], in a 50 μL volume on Days 0 and 14. Immediately following ID injection, suction was applied to the injection sites using the GeneDerm device (80 kPa, 30 s) using a 3‐mm cup opening. Blood was collected from the retro‐orbital plexus prior to vaccination and via cardiac puncture on Day 28 post‐CO_2_ euthanasia. Sera were stored at −20 °C for enzyme linked immunosorbant assay (ELISA) analysis against the SARS‐CoV‐2 spike protein as described [7]. Spleens were collected on Day 28 and immediately processed to harvest splenocytes for enzyme‐linked immunospot (ELISpot) analysis as described [7].

### *In vitro* transfection of HaCaT cells

A custom pVAX1‐based vector was designed using VectorBuilder (Chicago, IL) to include a strong cytomegalovirus (CMV) promoter that constitutively activates secreted embryonic alkaline phosphatase (SEAP) and was designated as pV1‐SEAP. To verify the performance of the new expression vector *in vitro*, adult human skin keratinocyte cells (HaCaT) (RRID:CVCL_0038; a gift from the lab of Francois Berthiaume at Rutgers University; originally obtained from Life Technologies, CA, USA which provides authenticated and mycoplasma‐free cells) were maintained using DMEM/F12 with 10% FBS and 1% anti‐anti (ThermoFisher, Waltham MA). HaCaT cells were transfected using FuGENE 6 (Madison, WI) and purified pV1‐SEAP DNA at a 3:1 ratio of FuGENE to DNA. Plasmid DNA (0.5, 1, 2, 4, and 6 μg) was incubated for 30 min at room temperature with 6 μL of FuGENE 6 and Opti‐MEM media (ThermoFisher, Waltham MA) to create DNA complexes. These complexes were then added dropwise to a 6‐well plate containing 250 000 HaCaT cells in DMEM/F12 and then incubated at 37 °C in 5% CO2 overnight. The media were changed to fresh DMEM/F12 24‐h post transfection, and supernatants were collected at baseline, after 24 h, and after 48 h, and stored at −20 °C. All experiments were performed using mycoplasma‐free cells. For SEAP determination, samples were thawed on ice, and SEAP was quantified using a Phospha‐Light SEAP Reporter Gene Assay System and plate reader (VarioSkan LUX, ThermoFisher, Waltham MA). Results were plotted and analyzed using graphpad prism.

### Perfusion of transfected HaCaT cells

Three wells of a 6‐well plate containing HaCaT cells (250 K cells) were transfected with pV1‐SEAP as above, with nontransfected wells serving as a control. Cells were perfused with DMEM/F12 media for 60 h at a flow rate of 0.5 mL·h^−1^ into the well with fractionated supernatant collected every two hours, as previously described [14]. SEAP concentrations were determined using the Phospha‐Light SEAP Reporter Gene Assay System (ThermoFisher, Waltham MA) and results analyzed for dynamic transgene activation dynamics using GraphPad Prism.

### *In vivo* transfection with pV1‐SEAP and analysis

On Week 1, Balb/c mice were sampled for a baseline reading of plasma SEAP levels by sampling 100 μL of whole blood. The plasma was separated then stored in −20 °C. At Week 2, the mice were injected ID with 20 μg pV1‐SEAP in a 50 μL injection volume in four regions in the dorsal area. Immediately following injection, suction was applied to each injection site using the GeneDerm device as above. At 24‐ and 72‐h postinjection, whole blood was collected, and plasma was separated to quantify circulating SEAP levels. The data were normalized to the background and to the total volume of the sample. Data were plotted and analyzed using graphpad prism.

## Results

### Optimization of suction‐mediated *in vivo* transfection in mice with long‐term transgene expression

Our prior studies showed that suction‐mediated *in vivo* transfection of plasmid DNA in rat skin was dependent on induced tissue strain and tension, which in turn depended on the applied suction pressure and opening diameter of the GeneDerm cup. Transfection was largely independent of the time that suction was applied [10, 15]. The marked laxity of mouse skin prevented successful extension of the current suction protocol to mice (data not shown), requiring modification of the methodology for injection and application of suction. To assess and optimize transgene expression of DNA plasmids following ID injection with suction‐mediated transfection in mice, expression from a luciferase‐encoded plasmid was studied.

To optimize the conditions for GeneDerm use in mice, we assessed multiple parameters. However, first it was required to optimize the method for mouse ID injections. Using a Mantoux injection technique, as we had done for other animals and humans, we found that creating a taut fold of skin through a gentle pinch and lift and then injecting into the superior aspect of skin along the raised fold yielded a fluid‐filled bleb akin to that observed in our other studies [10, 16] and indicating successful ID injection (Fig. 1).

**Fig. 2:**
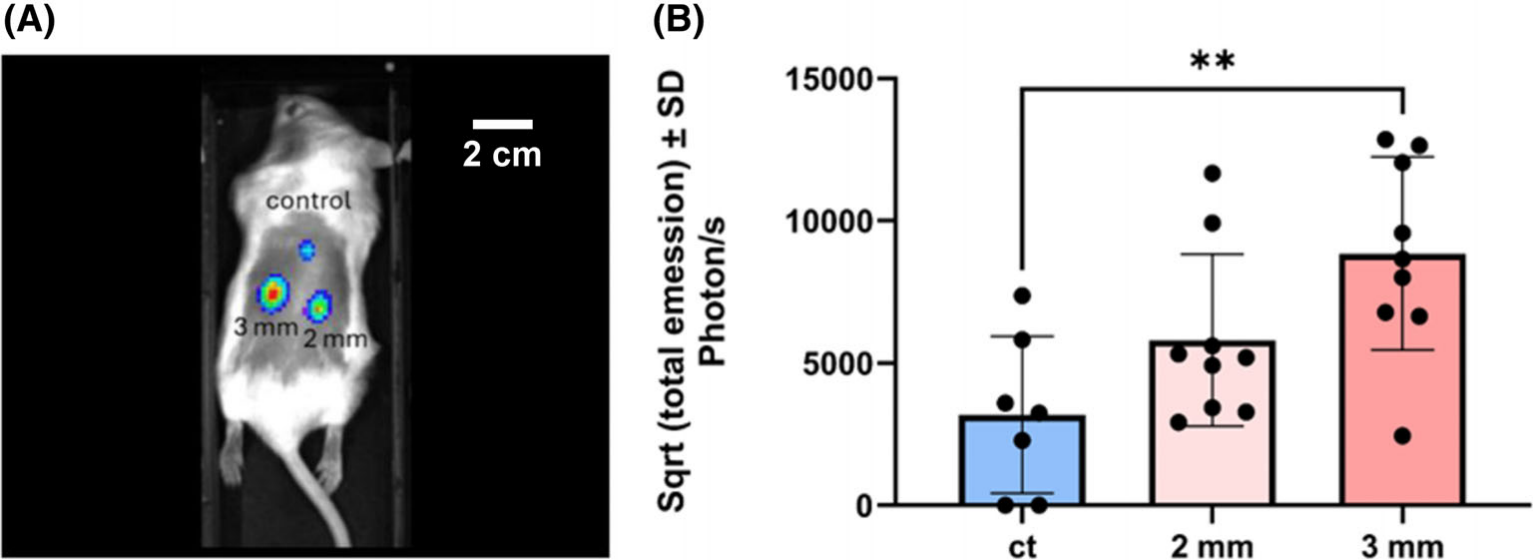
Luminescence expression in Balb/c mice using different cup diameters at various imaging time points. 50 μL of pGLS‐Luc2 was injected intradermally for all animals with or without suction pressure application; suction pressure was 80 kPa applied for 30 s using the GeneDerm device. (A) Representative luminescence image with 3‐s exposure time. (B) Luminescence quantification comparing 2‐ and 3‐mm cup diameters to injection only control 1‐day post treatment. Data represent mean ± SD. Statistics are presented as **P ≤ 0.01 by one‐way ANOVA followed by a Tukey's multiple comparisons test.

We next assessed the effects of the cup opening on the GeneDerm device through which suction is delivered to the skin. In early attempts to apply GeneDerm in mice, the 6‐mm‐diameter opening used in all other species [7, 8, 9, 10, 11] was too large for use in mice, as their comparatively looser skin was pulled into the cup and yielded no detectable luminescence (data not shown). Therefore, we used a cup opening diameter of either 2 or 3 mm. We assessed luciferase expression in Balb/c mice following injection of pGLS‐Luc2 either alone (as control) or followed by application of 80 kPa of suction for 30 s. Luminescence 24‐h postinjection was greater for conditions with applied suction, but it was only significantly different from injection‐only control when the 3‐mm cup opening was employed (Fig. 2). A qualitative assessment of the time course of luciferase expression using a 3‐mm cup opening (Fig. S1) showed that luminescence was detectable as early as 1‐h postinjection, was the highest from 1 through 9 days, and remained detectable up to 22 days postinjection. At each time point, pGLS‐Luc2 administered with suction yielded higher levels of luminescence than injection only. A similar preliminary assessment of suction‐mediated transfection in C57bl/6 mice was only minimally successful (data not shown) as luminescence detection is significantly decreased in this species due to their dark fur and skin, as reported by others [17].

**Fig. 3:**
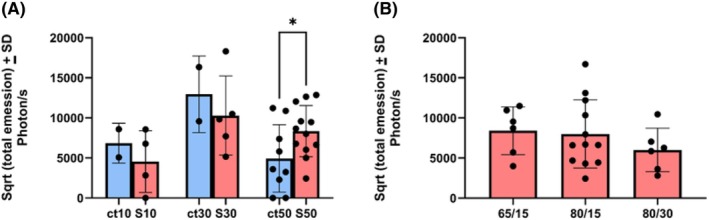
Effects of varying injection volumes, pressure, and time using Balb/C mice. (A) A comparison of injection only control, ‘ct’, at volumes of 10, 30, and 50 μL with suction pressure applied at 80 kPa for 30 s. (B) A 50‐μL injection was used to compare two pressures, 65 kPa and 80 kPa, applied for 15 or 30 s. Data represents mean ± SD of 3‐s exposure time of total luminescence emission. Statistics are presented as *P ≤ 0.05 by one‐way ANOVA followed by a Tukey's multiple comparisons test.

Finally, we assessed whether total injection volume or the strength and time of suction affected luciferase expression. Balb/c mice were injected with 25 μg of pGLS‐Luc2 as either a 10‐μL volume at a concentration of 2.5 μg·μL^−1^, a 30‐μL volume at a concentration of 0.83 μg·μL^−1^, or a 50‐μL at a concentration of 0.5 μg·μL^−1^. When suction was applied, only the 50‐μL injection showed statistically significant enhancement when compared with the injection‐only control (Fig. 3A). Base on this result we generally use the 50‐μL volume as the optimized injection volume. The preference of this volume is supported by our prior work of the colocalization of injected DNA to the region of greatest tissue strain (necessary for localized uptake) [16]. Finally, we found no significant difference in transgene expression when applying 65 kPa of suction for 15 s, 80 kPa for 15 s, or 80 kPa for 30 s with the GeneDerm device (Fig. 3B).

### Immune responses for mice following suction‐mediated transfection with GLS‐5310 COVID DNA vaccine

**Fig. 4:**
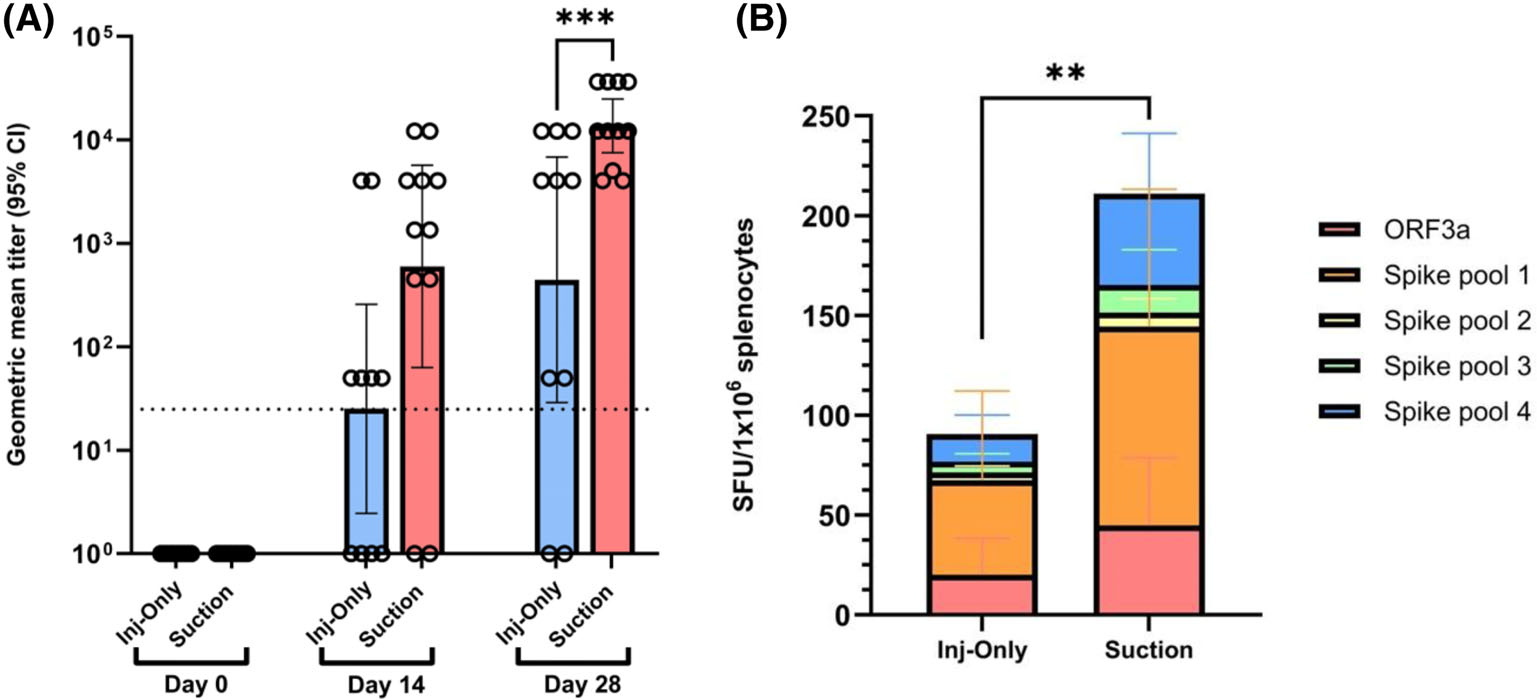
Immune responses to a SARS‐CoV‐2 DNA vaccine candidate using Balb/c mice. (A) Antibody responses of 50 μL injections of injection only control compared to 80 kPa pressure applied for 30 s with a 3‐mm‐diameter cup. (B) T‐cell responses quantified via spot forming units (SFU) by an ELISPOT assay to pools of peptides spanning the Spike and ORF3a proteins. Statistics are presented as **P ≤ 0.01 and ***P ≤ 0.001 by ordinary one‐way ANOVA followed by a Tukey's multiple comparisons test. Statistics in (B) are based on the total number of SFU by summing up contributions from the pools. Data represent mean ± SD.

As a further test of suction‐mediated *in vivo* transfection of mice for vaccination, we determined the immune response of Balb/c mice vaccinated with the GLS‐5310 SARS‐CoV‐2 DNA vaccine. Binding antibody titers against the SARS‐CoV‐2 spike protein for mice vaccinated with suction were approximately 1.5 log greater 14 days post second vaccination than injection‐only controls (*P* < 0.001; Fig. 4A). Additionally, all 10 suction‐treated animals seroconverted, versus 8 (80%) of injection‐only animals, two of which had only borderline responses. Vaccination with GLS‐5310 and GeneDerm suction demonstrated a broad‐based T‐cell response against both the spike and ORF3a proteins (Fig. 4B) that was more than 2‐fold higher (213.6 versus 90.7 for the total SFU summing up all pools; *P* ≤ 0.05) for animals vaccinated without suction.

### *In vitro* and *in vivo* measurement of SEAP expression

**Fig. 5:**
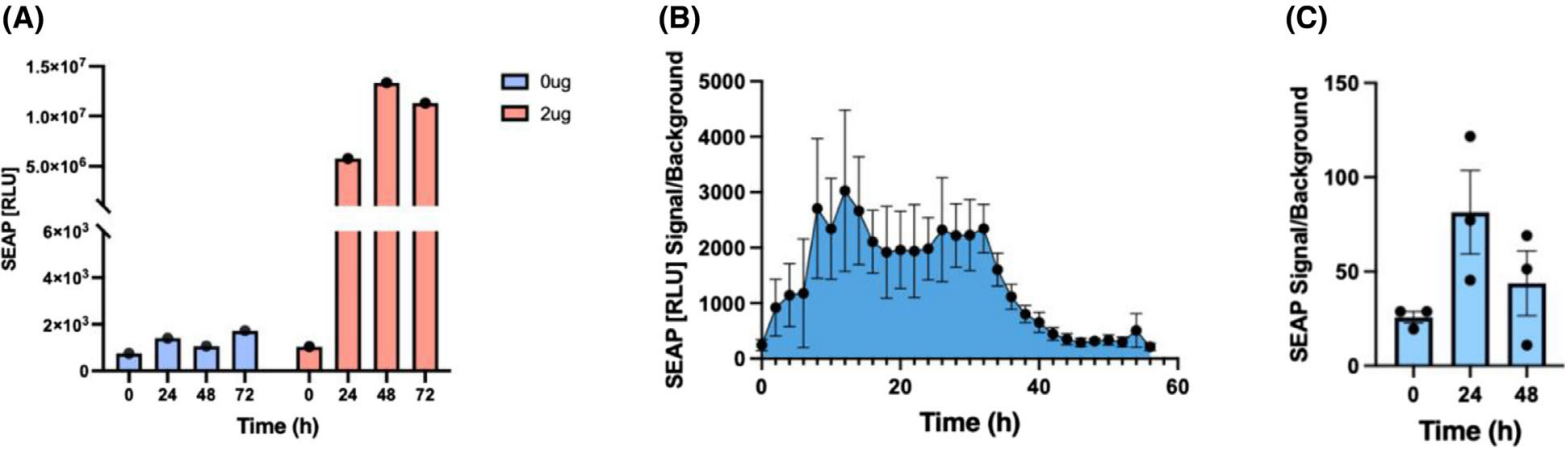
(A) *In vitro* transfection of HaCaT cells with 2 μg of pV1‐SEAP DNA compared to a control produces a 4‐log increase in SEAP production. (B) 60‐h kinetic perfusion assay of transfected HaCaT cells using 2 μg of pV1‐SEAP DNA illustrating the timescale dynamics of the transgene. Data represent mean ± SD. (C) Balb/c mice injected with 25 μg pV1‐SEAP DNA subcutaneously followed by 80 kPa of suction for 30 s with whole blood collection taken before the injection, 24 h post injection, and 48‐h post injection. The results were normalized to the background and volume of sample. A significant increase in SEAP production 24 h post transfection is present. Data represent mean ± SD.

We next assessed whether ID mediated transfection of mouse skin could result in production of a therapeutic protein. As a model, we constructed a novel SEAP expression plasmid, pV1‐SEAP. Prior to advancing to *in vivo* expression in mice, initial experiments were performed to optimize *in vitro* transfection of pV1‐SEAP and subsequent expression of SEAP in HaCaT cells by varying the amount of purified plasmid pV1‐SEAP DNA (Fig. S2). The most efficient condition was using 2 μg of pV1‐SEAP plasmid DNA, which yielded a four‐log increase in SEAP production relative to control cells (Fig. 5A and Supporting Information). We then determined the time course of SEAP expression in a kinetic assay perfusion system. Timescale dynamics showed a peak occurring at roughly 16 h with high levels of expression until 30 h followed by an exponential decay (Fig. 5B).

We then investigated whether and to what extent mice transfected with pV1‐SEAP would have detectable levels of expressed SEAP present in samples of circulating blood. Three Balb/c mice were injected with 25 μg of purified pV1‐SEAP DNA in a 50 μL volume, followed by application of suction using the GeneDerm device (80 kPa, 30 s) using a 3‐mm cup size. Peak values were threefold greater than pretreatment, with a peak at 24 h, decreasing to approximately 1.5 times baseline at 48 h (Fig. 5C).

## Discussion

Cutaneous suction‐mediated *in vivo* transfection was originally developed in a rat model, which was deemed a more suitable model because the mechanical and physical properties of rat skin are more comparable to that of humans [18, 19, 20, 21]. This similarity was evident, as the parameters used in rat skin were directly translated into use in human clinical trials without change [3, 7, 8]. However, mouse models are generally a more suitable model for early preclinical studies due to the availability of a wide range of supporting reagents, assays, and techniques [22, 23, 24, 25]. However, since the mechanical properties of mouse skin differ from rats and humans, adaptations of the method of ID injection, volume of material injected, and the diameter of the opening of the suction cup through which suction is transmitted were required. Indeed, parametric selection is the key to the success of cutaneous suction‐mediated *in vivo* transfection and immunogenicity. For example, Generotti et al. found that suction could enhance local transfection but not immunogenicity [26]. ID injection was successfully achieved using a smaller gauge needle (31 versus 28 gauge) and holding a fold of skin between the thumb and forefinger as demonstrated in Fig. 1. Injection volumes were decreased to 50 μL from 100 μL, and we used a suction cup with an opening diameter of 3 mm instead of the 6‐mm opening diameter used for other animals and humans. These parametric adaptations made important differences such that suction‐mediated transfection achieve both effectiveness and stability as shown, for example, in Fig. S1. On the other hand, as shown for rats [10], time‐of‐suction had minimal effect on transgene expression.

Note that although data points are limited, Fig. 2B does demonstrate an approximate linear correlation between expression signal and cup size. This is not surprising and is actually consistent with our prior work on a rat model where via extensive parametric study we found that expression strength is correlated linearly with the product of suction pressure and cup diameter for cases above the threshold of 35 kPa [15]. This result was interpreted as that the tissue tension and strain are the main factors driving endocytosis and mediating expression strength [10]. While we expect the same trend in a mouse model, a similar parametric study is required to provide full quantitative validation. Discussions on possible molecular mechanisms are found in our prior work [10, 15].

To validate the results obtained after transfection with a vector encoding luciferase, we immunized mice with the GLS‐5310 SARS‐CoV‐2 DNA vaccine. Suction significantly enhanced both the antibody and the cellular immune responses compared to the injection‐only group, consistent with our prior studies in rats and hamsters [7, 10].

Finally, we showed that suction‐mediated *in vivo* transfection of the skin yielded systemic levels of an expressed transgene. We first established that pV1‐SEAP, a pVAX1 expression vector with a constitutive CMV promoter, transfected into human HaCaT skin cells *in vitro* yields high levels of SEAP expression. *In vivo* transfection of mice using suction showed transgene activation dynamics similar to the *in vitro* model. Considering the fact that the serum half‐life of SEAP is approximately two hours [27], our data are consistent with constitutive production of SEAP *in vivo*.

## Conclusion

In conclusion, in this study, we have established and optimized an intradermal suction‐mediated DNA delivery method for mice. We additionally demonstrate that *in vivo* suction‐mediated transfection of a COVID‐19 DNA vaccine was highly immunogenic and that transfection of a SEAP expressing plasmid yielded high levels of circulating protein detected in blood with a pharmacodynamic profile similar to that seen *in vitro*.

## Conflict of interest

Emran O. Lallow, Melissa Gulley, Sagar Kudchodkar, Christine C. Roberts and Joel N. Maslow are employees of GeneOne Life Science Inc. The remaining authors declare no conflict of interest.

## Author contributions

EOL, DIS, BP, LKD, HL, and JNM conceived and designed the experiments for this study. EOL, IB, YM, LO, MG, SK, and NCJ performed the experiments. HL, JNW, LKD, DIS, and BP provided resources and supervision. EOL and IB wrote the original concept and manuscript, and HL, JNW, CCR, LKD, DIS, BP, and YM reviewed and edited it.

## Supporting information


**Fig. S1.** (A) Representative luminescence images from 1 h to 22 days. (B) Quantified luminescence level (control ‐ blue and left; suction‐red and right). Data represents mean ± SD.
**Fig. S2.** Optimization of pV1‐SEAP plasmid DNA transfection *in vitro*.

## Data Availability

The data that support the findings of this study are available from the corresponding author upon reasonable request.
