# Supplementary material for: Delivery of DNA‐encoded vaccines and proteins in mice using cutaneous suction‐mediated transfection
Source: FEBS Open Bio. 2026 Jul 9:10.1002/2211-5463.70306. Online ahead of print. doi: 10.1002/2211-5463.70306 (PMC13399011; doi:10.1002/2211-5463.70306)
Supplement: Supplementary file 1 — Fig. S1. (A) Representative luminescence images from 1 h to 22 days. (B) Quantified luminescence level (control ‐ blue and left; suction‐red and right). Data represents mean ± SD. Fig. S2. Optimization of pV1‐SEAP plasmid DNA transfection in vitro. [file FEB4-9999-0-s001.pdf]

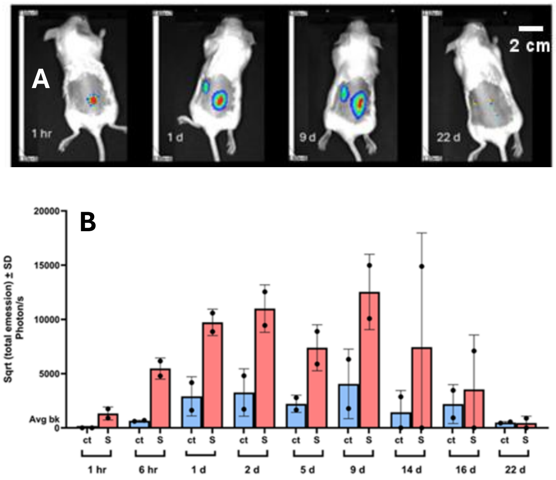

Figure S1: (A) Representative luminescence images from 1 hr to 22 days. (B) Quantified luminescence level (control- blue and left; suction-red and right). The image threshold is automatically adjusted to show the best signal comparison between the injection-only control cases and the suction cases. Data represents mean±SD.

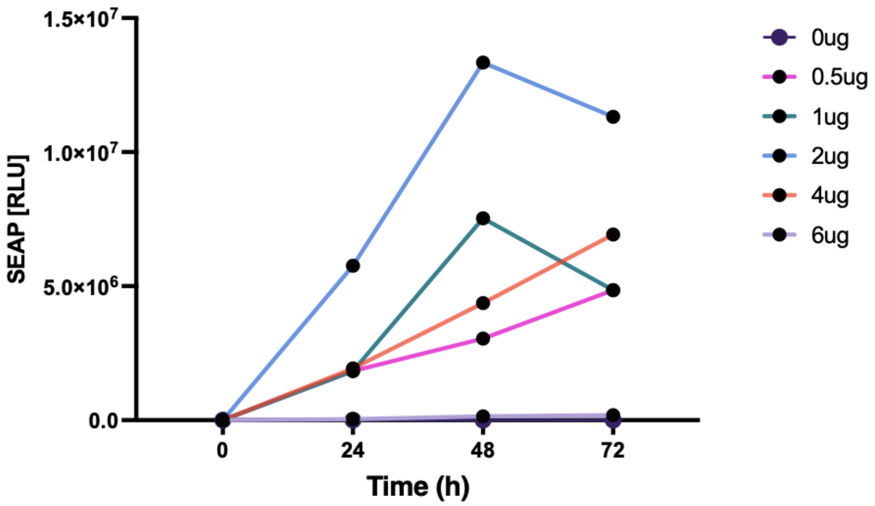

Figure S2: Optimization of pV1-SEAP plasmid DNA transfection in vitro. HaCaT cells were transfected using FuGENE (Madison, WI) in a 3:1 FuGENE to DNA ratio with 0-6 µg of purified plasmid DNA. Supernatant samples were collected at 0-, 24-, 48-, and 72-hours post transfection and analyzed using the Phospha-Light SEAP Reporter Gene Assay System (ThermoFisher, Waltham MA) and GraphPad Prism to plot the dynamic transgene activation dynamics.
